# Helicobacter Pylori-Associated Gastritis in Middle Eastern Patients: A Case Series

**DOI:** 10.7759/cureus.39145

**Published:** 2023-05-17

**Authors:** Zahid Khan, Amresh Gul

**Affiliations:** 1 Acute Medicine, Mid and South Essex NHS Foundation Trust, Southend on Sea, GBR; 2 Cardiology, Bart’s Heart Centre, London, GBR; 3 Cardiology and General Medicine, Barking, Havering and Redbridge University Hospitals NHS Trust, London, GBR; 4 Cardiology, Royal Free Hospital, London, GBR; 5 General Practice, Lifeline Hospital, Salalah, OMN

**Keywords:** helicobacter pylori and celiac disease, treatment of helicobacter pylori infection, helicobacter pylori resistance, helicobacter pylori and autoimmunity, helicobacter pylori eradication, chronic atrophic gastritis, generic antibiotics, coeliac disease, middle-eastern populations, proton pump inhibitors (ppis)

## Abstract

Helicobacter pylori (H. pylori) infection is a common chronic infection responsible for upper gastrointestinal symptoms such as indigestion, belching, heartburn, and abdominal fullness along with nausea and vomiting. It is considered a transmissible infection, although the exact chain of transmission is not clear. H. pylori-associated infection is a major pathogenic factor for gastroduodenal ulcers and gastric carcinoma in most patients, which can be prevented with eradication therapy. Transmission of the bacterium occurs mainly between family members and during childhood. Others may remain asymptomatic or may present with atypical symptoms such as headache, fatigue/weakness, anxiety, and bloating. We present five interesting cases of H. pylori-positive patients who had variable presentations and were successfully treated with first-line therapy and salvage therapy.

## Introduction

Helicobacter pylori (H. pylori) is a common gram-negative bacterium that can colonise the human stomach and cause chronic gastritis, peptic ulcer disease, mucosa-associated lymphoid tissue lymphoma, and gastric cancer [1]. H. pylori infection is one of the most common chronic infections worldwide, with an estimated prevalence of 50% in developing countries and 10-20% in developed countries [2]. The World Health Organization (WHO) has identified H. pylori as a global priority pathogen due to its increasing prevalence and the emergence of antibiotic resistance [3]. Infection with H. pylori induces a persistent immune response. Because the organism is capable of numerous adaptations to prevent immune detection, its clearance by the body is never complete. The resulting sustained inflammatory processes in the stomach cause a reduction in the population of somatostatin-producing D cells [1,4]. This causes a subsequent rise in gastrin secretion followed by an increase in gastric acid release, which may lead to peptic ulceration in some patients [5,6]. A sedentary lifestyle and consumption of red meat and junk food have been considered common risk factors for the infection. In addition, other risk factors include poor hygiene, poor socioeconomic status, poor sanitation, overcrowding, consumption of contaminated water and food, and bacterial infection within the household that can get transferred between family members [7]. We present five cases of positive H. pylori infection treated with first-line therapy and salvage therapy following the failure of first-line and second-line therapies.

## Case presentation

Case 1

A 42-year-old male, who was a known case of asthma, chronic gastritis, and asymptomatic hyperuricemia, presented with complaints of persistent pain in the epigastrium region, indigestion, belching, and fullness for the past few days. He also reported experiencing headaches, especially in the occipital region, and weakness of the lower limbs, which were perhaps unrelated symptoms. Previously, he had been successfully treated for H. pylori infection in 2022 with a combination of amoxicillin, clarithromycin, and esomeprazole for two weeks. He had also been a heavy smoker for the last 15 years and smoked >20 cigarettes daily. The clinical examination was unremarkable; his blood pressure was 134/90 mmHg and he was overweight with a body mass index (BMI) of 29 kg/m^2^. His urea breath test (UBT) was positive with a value of 945 disintegrations per minute (DPM), and the rest of the investigations were unremarkable. Hence, he was treated with a combination of metronidazole, clarithromycin, and pantoprazole twice a day for two weeks. Subsequently, he became symptom-free and his UBT count dropped to 25 DPM.

Case 2

A 21-year-old male came to our hospital complaining of upper abdomen pain for the past few weeks along with abdominal fullness, especially after meals. He also reported a feeling of heaviness in the head and weakness in the lower limbs. Moreover, he had been apprehensive and had a feeling of heaviness in his chest. He consumed camel meat at least a couple of times a day and ate junk food. Clinical examination revealed tenderness in the epigastrium on deep palpation but was otherwise unremarkable. His blood reports were within normal range, and an ultrasound of the abdomen did not reveal any abnormality; however, his UBT was positive with more than 714 count DPM. As a result, he was treated with a combination of amoxicillin, clarithromycin, and pantoprazole twice a day for two weeks. Despite this, he was still experiencing the same symptoms, and hence he was treated with alternative triple therapy, including metronidazole. On follow-up visits, the symptoms had alleviated, and the follow-up UBT test returned negative.

Case 3

A 34-year-old female presented with a 10-day history of upper abdominal fullness, nausea, and indigestion. The pain was mild to moderate in severity, localised, not referred, and burning in character. It was associated with nausea and regurgitation of the food, especially after dinner because she went to bed immediately after dinner. Furthermore, she added that her diet predominantly consisted of meat, fish, and junk food, especially from restaurants. Her medical history was unremarkable; however, she had experienced iron deficiency anaemia a few years ago. Examination showed an anaemic individual, who was concerned and anxious about having a bacterial infection of the stomach as her husband had tested positive a few weeks ago and was treated accordingly. Her BMI was 31 kg/m^2^. Her hemoglobin was 9.1, MCV was 62.4, the iron level was 2.2 mg/dl, ferritin was 4.56 mg/dl, potassium level was 4.2 mmol, sodium was 142 mmol, and UBT was positive with a count of 1942. Consequently, she was started on first-line triple therapy with a combination of metronidazole, clarithromycin, and esomeprazole twice a day for two weeks. However, her symptoms persisted, and she was advised to undergo an endoscopy, but she was not ready to do any invasive procedures. She was then successfully treated with salvage therapy, including levofloxacin, clarithromycin, and esomeprazole twice a day for 10 days. She was also advised about diet and lifestyle modification.

Case 4

A 43-year-old male, a heavy smoker, came to our hospital with atypical symptoms such as headaches, heaviness, stress, apprehension, and weakness of the legs for the past few weeks. On further questioning, he reported gastrointestinal symptoms such as a burning sensation in the epigastrium region, indigestion, and belching. Like the other two patients, he consumed an enormous amount of meat, even for breakfast at times; he did not engage in any physical exercise, consumed a lot of coffee, and often dined outside, particularly junk food. On examination, his BMI was 33 kg/m^2^, and he had a blood pressure of 145/83 mmHg; the examination was otherwise unremarkable, including his mental state examination. Subsequently, the blood reports showed hemoglobin of 13.2 mg/dl, LDL of 130 mg/dl, TG of 198 mg/dl, total cholesterol of 212 mg/dl, HDL of 41 mg/dl, and slightly elevated liver enzymes. His H. pylori antigen test was positive. On the following day, his UBT showed a value of 1674 DPM (normal level: less than 200), and an ultrasound of the abdomen confirmed grade 2 fatty liver disease. Hence, he was treated with a combination of amoxicillin, clarithromycin, and omeprazole twice a day for two weeks. In addition, he was advised to reduce his weight and avoid the consumption of oily and junk food. On follow-up visits, the patient's symptoms had abated and the follow-up UBT was negative with a count of 145 DPM.

Case 5

A 42-year-old male, who was a known case of diabetes, hypertension, gout, and chronic gastritis presented with complaints of regurgitation, heartburn, burping, and indigestion for the past few months. He worked in a remote area and ate locally at restaurants and consumed meat most of the time. His medications encompassed metformin, sitagliptin, amlodipine, valsartan, and rosuvastatin along with allopurinol. In addition, he also took ibuprofen 400 mg for pain. Examination revealed blood pressure of 141/88 mmHg and pulse rate of 87 bpm; he was overweight with a BMI of 31 kg/m^2^. His blood reports showed elevated components of lipids, raised liver enzymes, and uric acid of 7.5 mg/dl. His C-13 UBT was positive with a count of 112 (reference range: C≤40 negative, C≥50 positive, C=41-49 borderline). Consequently, he was treated with a combination of metronidazole, clarithromycin, and pantoprazole twice a day for two weeks. On the follow-up visit, while the patient's symptoms had improved, he had been experiencing them intermittently. Therefore, he was advised to continue with acid suppression therapy for the next two weeks. Subsequently, his UBT count dropped to 002. He was also advised to adopt lifestyle modification along with dietary changes.

The lab values and symptom comparison of the patients are presented in Tables 1, 2 respectively.

**Table 1:** Lab values for all five patients ALT: alanine aminotransferase; AST: aspartate aminotransferase; WBC: white blood cell count; CRP: C-reactive protein; ESR: erythrocyte sedimentation rate

Blood tests	Case 1	Case 2	Case 3	Case 4	Case 5	Normal range
Hemoglobin	13.2	14.6	9.1	15.2	13.9	13-18 gm/dl
WBC count	10.4	9.56	5.3	11.1	6.5	3.5-11 × 10^9^/L
CRP	03	01	07	16	02	<5 mg/L
ESR	02	04	01	13	02	0-2 mg/L
Sodium	134	141	139	145	137	135-145 mmol/L
Potassium	3.9	4.2	3.7	4.9	3.6	3.5-5.1 mmol/L
Urea	4.7	3.1	4.1	5.0	3.6	2.9-8.2 mmol/L
Creatinine	76	67	88	65	83	66-112 umol/L
ALT	45	56	43	93.4	64	<40 IU/L
AST	36	45	65	110	76.2	<40 IU/L
Fasting blood glucose	92	86	93	103	97	<100 mg/dl
Urea breath test	945	714	1942	1674	112	<200
C-13	123	-	-	118	112	<40

**Table 2:** Symptom comparison among the patients

Case number	Symptoms
Case 1	Persistent pain in the epigastrium, indigestion, belching and fullness, occipital headache, and weakness of the lower limbs
Case 2	Upper abdomen pain, abdominal fullness, heaviness of head, and weakness of the lower limbs, apprehensive and anxious, described the condition as "having something over my chest and neck region"
Case 3	Upper abdominal fullness, inability to eat more, nausea and indigestion, nausea and regurgitation of the food
Case 4	Headache, heaviness, stress, apprehension, weakness of the legs, burning sensation in the epigastrium, indigestion, and belching
Case 5	Regurgitation, heartburn, burping, and indigestion

## Discussion

H. pylori infection is commonly found in Middle Eastern countries, in particular Oman. The prevalence of infection is higher in people who are young, middle-aged, those who consumed plenty of camel meat, smokers, and those who led sedentary lifestyles. The likelihood of infection is most strongly related to living conditions in childhood (when acquisition usually occurs). Infection is more commonly found in family members of an infected person and in people with a family history of peptic ulcer disease or gastritis [8].

In younger adult patients (generally under 50 years of age, but younger if the patient is from a higher-risk region, e.g., East Asia, the Mediterranean region) with dyspepsia but no alarming symptoms, the "test-and-treat" strategy (using a noninvasive diagnostic test for H. pylori) has been shown to be cost-effective. It is just as (or more) likely to result in symptom relief as endoscopy or empirical treatment. All people infected with H. pylori develop active chronic gastritis, but there is an inconstant relationship between the presence of H. pylori gastritis and symptoms. Thus, gastritis is a pathological rather than a clinical diagnosis [9].

The usual first-line test is UBT, while others are faecal antigen testing, serology, or invasive tests based on endoscopic biopsy such as rapid urease test or histology. Prompt endoscopy is required if the patient has red flag symptoms such as weight loss, anaemia, melena, lymphadenopathy, or persistent symptoms despite the triple therapy. Antibiotics should be halted for at least four weeks; proton pump inhibitors should be withheld for at least one week (preferably two weeks) before a urea breath or faecal antigen test for H. pylori, to minimise the chance of false-negative results.

Eradication triple therapy should be commenced for patients who are found to be infected with H. pylori. However, eradicating H. pylori is not appropriate in some patients; the emphasis should be on the patient’s goals of care, the potential adverse effects of eradication therapy, and whether eradication will significantly improve the patient’s quality of life. The first-line H. pylori eradication therapy involves clarithromycin-based triple therapy. If first-line therapy fails, clarithromycin resistance is likely, and hence alternative (salvage) therapy is needed [10,11]. Salvage therapy is only occasionally guided by culture and susceptibility testing obtained from an endoscopic biopsy. Primary H. pylori resistance to levofloxacin has not yet been observed to be a problem in any region of the world. H. pylori resistance to amoxicillin is high in Oman, and H. pylori resistance to tetracycline is almost nonexistent [11].

Eshraghian (2014) reported a high prevalence of asymptomatic H. pylori patients in the Middle Eastern region [12]. The overall reported prevalence of H. pylori infection in Iran ranged from 30.6% to 82%, while it ranged from 22% to 87.6% in other Middle Eastern countries. Ayoola et al. (2004) found that 60.1% of chronic gastritis and 62.8% of duodenal ulcer patients diagnosed by endoscopy had H. pylori infection [13]. The rate of H. pylori infection was higher in patients with duodenal ulcers compared to those with normal endoscopic findings. They also reported a difference in the prevalence of the disease between urban and rural populations, which they believed could be due to the use of non-steroidal anti-inflammatory drugs (NSAIDs) and environmental factors.

## Conclusions

H. pylori infection remains a significant cause of morbidity worldwide. All of our five patients were obese, consumed plenty of meat and spicy and oily food along with junk food, especially at restaurants, led sedentary lifestyles, did not engage in physical exercise, dined late at night, and were smokers. Early intervention with triple therapy along with dietary changes is the cornerstone of the treatment of this condition. Further cross-sectional studies would be useful to assess the prevalence of the disease and the use of eradication therapy among both rural and urban populations.
